# Incidence of symptomatic *Borrelia burgdorferi *sensu lato infection in Romania, 2018−2023

**DOI:** 10.1186/s13071-024-06449-5

**Published:** 2024-09-05

**Authors:** Frederick J. Angulo, Julia Olsen, Veronica Purdel, Mihaela Lupșe, Adriana Hristea, Violeta Briciu, Emily Colby, Andreas Pilz, Kate Halsby, Patrick H. Kelly, Gordon Brestrich, Jennifer C. Moïsi, James H. Stark

**Affiliations:** 1 Vaccines and Antivirals Medical Affairs, Pfizer US Commercial Division, 500 Arcola Road, Collegeville, PA 19426 USA; 2 Pfizer Romania SRL, Vaccines, Willbrook Platinum Business & Convention Center Sos, București–Ploiești No. 172–176, Building B, Stage 5, Sector 1, Bucharest, 013686 Romania; 3 Department of Infectious Diseases, “Iuliu Hatieganu” University of Medicine and Pharmacy, Cluj-Napoca, Romania; 4 University of Medicine and Pharmacy Carol Davila, Bucharest, Romania; 5 Pfizer Corporation Austria, Vaccines and Antivirals Medical Affairs, Floridsdorfer Hauptstraße 1, 1210 Vienna, Austria; 6 Vaccines and Antivirals Medical Affairs, Dorking Road, Tadworth, Surrey, KT20 7NY UK; 7 Pfizer Pharma GmbH, Vaccines and Antivirals Medical Affairs, Friedrichstraße, 110-10117 Berlin, Germany; 8 Vaccines and Antivirals Medical Affairs, 23 Avenue du Docteur Lannelongue, 75014 Paris, France; 9 Vaccines and Antivirals Medical Affairs, Pfizer US Commercial Division, 1 Portland Street, Cambridge, MA 02139 USA; 10 Present Address: Hologic, Inc, Marlborough, MA 01752 USA

**Keywords:** Disease burden, Epidemiology, Lyme borreliosis, Tick-borne disease

## Abstract

**Background:**

Lyme borreliosis (LB), caused by *Borrelia burgdorferi *sensu lato (Bbsl), is the most common tick-borne disease in Europe. Although public health surveillance for LB has been conducted in Romania since 2007, the extent of under-detection of Bbsl infections by LB surveillance has not been estimated. We therefore estimated the under-detection of symptomatic Bbsl infections by LB surveillance to better understand the LB burden in Romania.

**Methods:**

The number of incident symptomatic Bbsl infections were estimated from a seroprevalence study conducted in six counties (population 2.3 M) and estimates of the symptomatic proportion and duration of persistence of anti-Bbsl immunoglobulin G (IgG) antibodies. The number of incident symptomatic Bbsl infections were compared with the number of surveillance-reported LB cases to derive an under-detection multiplier, and then the under-detection multiplier was applied to LB surveillance data to estimate the incidence of symptomatic Bbsl infection from 2018 to 2023.

**Results:**

We estimate that there were 1968 individuals with incident symptomatic Bbsl infection in the six counties where the seroprevalence study was conducted in 2020, compared with the 187 surveillance-reported LB cases, resulting in an under-detection multiplier of 10.5 (i.e., for every surveillance-reported LB case, there were 10.5 symptomatic incident Bbsl infections). The incidence of symptomatic Bbsl infection in the six counties was 86.9/100,000 population in 2023, similar to the incidence in 2018−2020 (86.0) and higher than in 2021−2022 (40.3).

**Conclusions:**

There is a higher incidence of symptomatic Bbsl infection than is reported through public health surveillance for LB in Romania. Additional efforts are needed to strengthen disease prevention and address the important public health problem of LB.

**Graphic Abstract:**

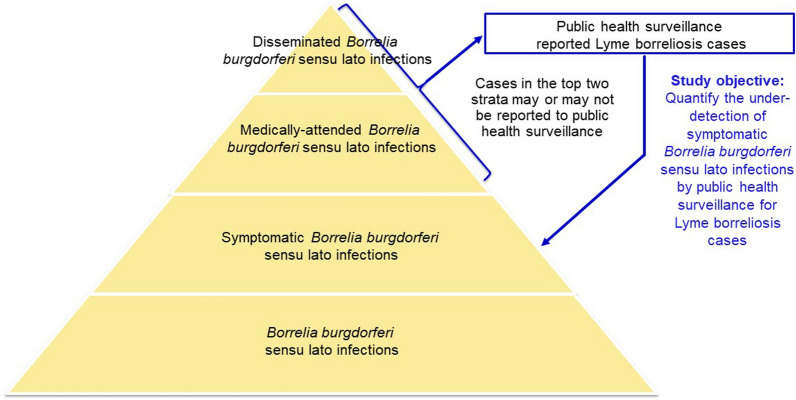

## Background

Lyme borreliosis (LB), the most common tick-borne disease in Europe, is caused by the spirochete *Borrelia burgdorferi *sensu lato (Bbsl), which is transmitted to humans predominately by the bite of an *Ixodes* tick [1]. Patients with LB most commonly present for medical care with a localized skin disease in the form of erythema migrans (EM). However, Bbsl infections can also disseminate and result in disseminated LB including Lyme neuroborreliosis, arthritis, and carditis. LB is endemic in parts of Romania, and elsewhere in Europe [2, 3]. Many European countries conduct public health surveillance for LB, but differences in case definitions, diagnostic practices, and laboratory methods complicate efforts to estimate LB disease burden using surveillance data [2, 3].

Public health surveillance for LB in Romania began in 2007 with statutory reporting of LB cases by physicians to county health officers, who then report LB cases to the National Institute of Public Health (NIPH). Surveillance-reported LB cases include clinician-diagnosed cases with a laboratory confirmation (i.e., confirmed cases) and clinician-diagnosed cases without a laboratory confirmation (i.e., probable cases). NIPH publishes annual LB surveillance reports, with data available at the county level. The incidence of surveillance-reported LB per 100,000 population per year (PPY) was 2.3/100,000 from 2010 to 2023; the lowest incidence per 100,000 PPY was in 2021, which coincided with the coronavirus disease 2019 (COVID-19) pandemic [4].

**Fig. 1:**
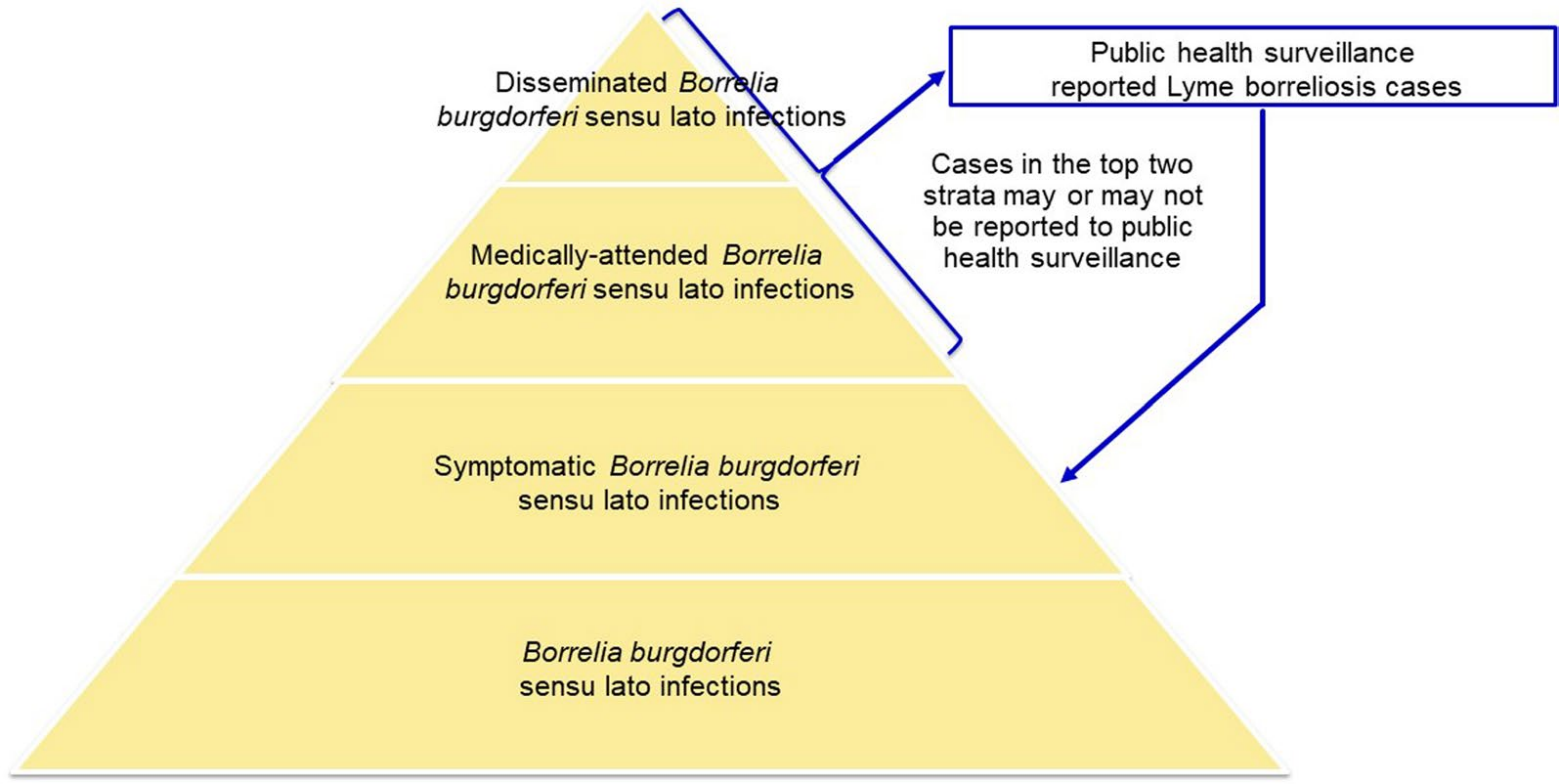
Pyramid of *Borrelia burgdorferi* sensu lato infections

Surveillance-reported LB cases represent only a portion of symptomatic Bbsl infections (Fig. 1). There are several reasons why an individual with a symptomatic Bbsl infection may not be detected by public health surveillance [5]. The extent of under-detection of symptomatic infections by public health surveillance can be estimated through the derivation of an under-detection multiplier, which is obtained by comparing the estimated number of incident symptomatic infections derived from a seroprevalence study with the number of surveillance-reported cases [6–8]. Studies have indicated that there is under-detection of LB cases by public health surveillance in Romania, but the extent of this under-detection has not previously been estimated [9, 10]. The objective of this study was to quantify the under-detection of Bbsl infection by LB surveillance and thereby estimate the incidence of symptomatic Bbsl infection in Romania.

## Methods

We estimated the incidence of symptomatic Bbsl infection in Romania using a seroprevalence-based approach. We utilized a systematic literature review of studies published from 2005 to 2020 [11] and conducted a PubMed literature search of articles published from 2021 to 2023 to identify seroprevalence studies conducted in Romania that measured the prevalence of anti-Bbsl IgG antibodies in the general population. Public health LB surveillance data, nationally and by county, were retrieved from NIPH annual reports (https://www.insp.gov.ro). The population of Romania was 19 million in 2020. The country is divided into 41 counties and the municipality of Bucharest.

We derived two additional estimates to calculate the incidence of symptomatic Bbsl infection from seroprevalence data: an estimate of the symptomatic proportion among individuals with an incident Bbsl infection and an estimate of the duration of persistence of anti-Bbsl IgG antibodies in Bbsl-infected persons. To derive these estimates, we conducted a PubMed literature search to identify follow-up studies conducted in Europe of Bbsl-infected individuals published from 1990 to 2023. To estimate the symptomatic proportion, an individual with an incident Bbsl infection was defined as an individual who sero-converted from anti-Bbsl IgG-negative to IgG-positive. To estimate the duration of persistence of anti-Bbsl IgG antibodies, the duration was defined as the time from Bbsl infection to sero-reversion from anti-Bbsl IgG-positive to IgG-negative.

The prevalence of anti-Bbsl IgG antibodies (P) reported in identified seroprevalence studies was used to estimate the number of individuals with an incident Bbsl infection (I) using an estimate of the duration of persistence of anti-Bbsl IgG antibodies (D) and the formula I = P/D. The incidence of symptomatic Bbsl infection (per 100,000 PPY) was then calculated using mid-year population estimates and an estimate of the symptomatic proportion. The incidence of symptomatic Bbsl infection was compared with the incidence of surveillance-reported LB cases in the same year that the specimens were collected in the seroprevalence studies to derive the under-detection multiplier for symptomatic Bbsl infections by public health surveillance. The under-detection multiplier was then applied to the incidence of surveillance-reported LB cases in 2018−2023 to derive an estimate of the incidence of symptomatic Bbsl infection in 2018−2023.

## Results

**Fig. 2:**
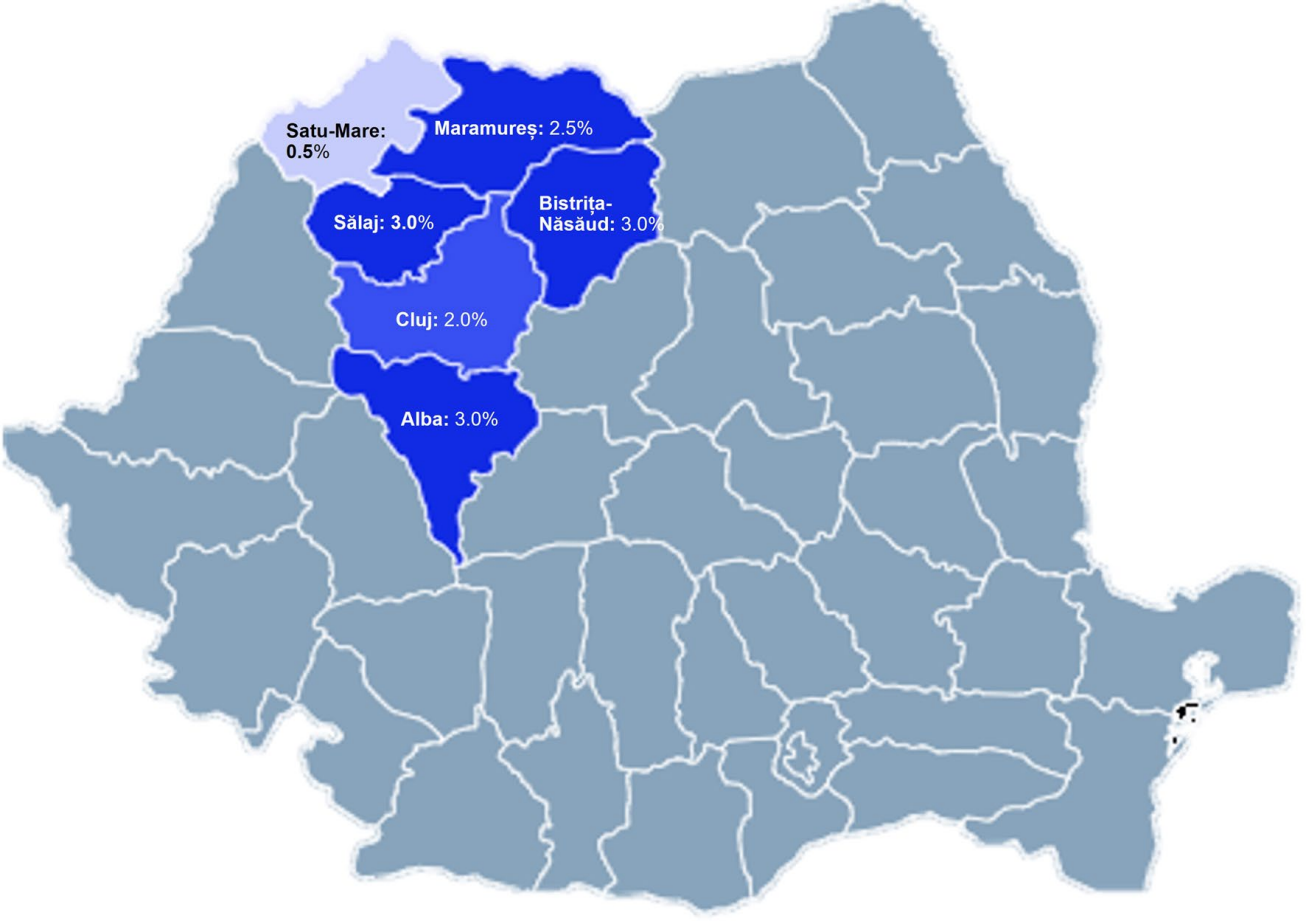
Seroprevalence of anti-*Borrelia burgdorferi* sensu lato IgG antibodies from the 2019–2020 seroprevalence study in six counties in Romania

We identified one general population LB seroprevalence study conducted in Romania [12]. The study, by Kalmár et al., collected serum samples from November 2019 to September 2020 from 1200 adult blood donors in six counties in northwestern and central Romania: Alba, Bistrița-Năsăud, Cluj, Maramureș, Sălaj, and Satu-Mare. The population in the six counties was 2,312,385 in 2020, which was 12.2% of the population in Romania. Samples were tested for anti-Bbsl IgG antibodies using the enzyme-linked immunosorbent assay (ELISA) test recomWell Borrelia kit (Mikrogen Diagnostik, Germany), with ELISA-positive or equivocal samples further tested using the recomLine Borrelia kit (Mikrogen Diagnostik, Germany). Samples positive using the recomLine test were considered positive for anti-Bbsl IgG antibodies. The prevalence of anti-Bbsl IgG antibodies in the six counties in 2019–2020 was 2.3% (95% CI 1.6%−3.4%); stratified by county, the prevalence ranged from 0.5% to 3.0% (Fig. 2).

The literature search identified five follow-up studies that involved individuals with an incident Bbsl infection who were interviewed about symptoms [13–17]. All were cohort studies; four studies enrolled individuals bitten by a tick and one study enrolled individuals at high risk of being bitten by a tick. The studies identified 110 individuals with an incident Bbsl infection. The pooled estimate of the symptomatic proportion in the five studies was 37% (95% CI 28%−46%). The search also identified eight follow-up studies that reported on the duration of persistence of anti-Bbsl IgG antibodies [18–25]. Six were follow-up studies of individuals with anti-Bbsl IgG antibodies identified when they sought medical care and two were follow-up studies of individuals with anti-Bbsl IgG antibodies identified by general population seroprevalence studies. The studies included 3352 individuals with anti-Bbsl IgG antibodies. The range in the estimated time for 50% of the individuals with anti-Bbsl IgG antibodies to serorevert to IgG-negative was 0.4−33.3 years. Given the range in median time of the duration of persistence of IgG antibodies in Bbsl-infected individuals in the eight studies, we used a 10-year duration of persistence of anti-Bbsl IgG antibodies.

Using the anti-Bbsl IgG prevalence of 2.3% from the seroprevalence study conducted in Romania, a symptomatic proportion of 37%, and persistence of anti-Bbsl IgG antibodies of 10 years, there were an estimated 1968 individuals with symptomatic Bbsl infection in the six counties in Romania in 2020 and an incidence of symptomatic Bbsl infection of 85.1/100,000 PPY (Table 1). There were 187 surveillanc-reported LB cases in the six counties in 2020 (an incidence of surveillance-reported LB of 8.1/100,000 PPY). Therefore, the under-detection multiplier of symptomatic Bbsl infections by public health surveillance was 10.5 (i.e., for each surveillance-reported LB case, there were 10.5 symptomatic Bbsl infections) in the six counties in 2020.

**Table 1:** Estimates of the number of incident symptomatic *Borrelia burgdorferi* sensu lato infections and incidence (per 100,000 population per year) of symptomatic *Borrelia burgdorferi* sensu lato infection in six counties (Alba, Bistriţa-Năsăud, Cluj, Maramureş, Sălaj, and Satu-Mare) in Romania, 2020

County	Sero-prevalence in 2020^a^ (%)	Population in 2020	Prevalent symptomatic Bbsl infections in 2020	Incident symptomatic Bbsl infections in 2020	Incidence of symptomatic Bbsl infections in 2020
Alba	3.0	324,071	9722	360	111.0
Bistriţa-Năsăud	3.0	278,033	8341	309	111.0
Cluj	2.0	709,872	14,197	525	74.0
Maramureş	2.5	458,927	11,473	425	92.5
Sălaj	3.0	209,982	6299	233	111.0
Satu-Mare	0.5	331,500	1658	61	18.5

Bbsl, *Borrelia burgdorferi* sensu lato

^a^Kalmár et al.

**Fig. 3:**
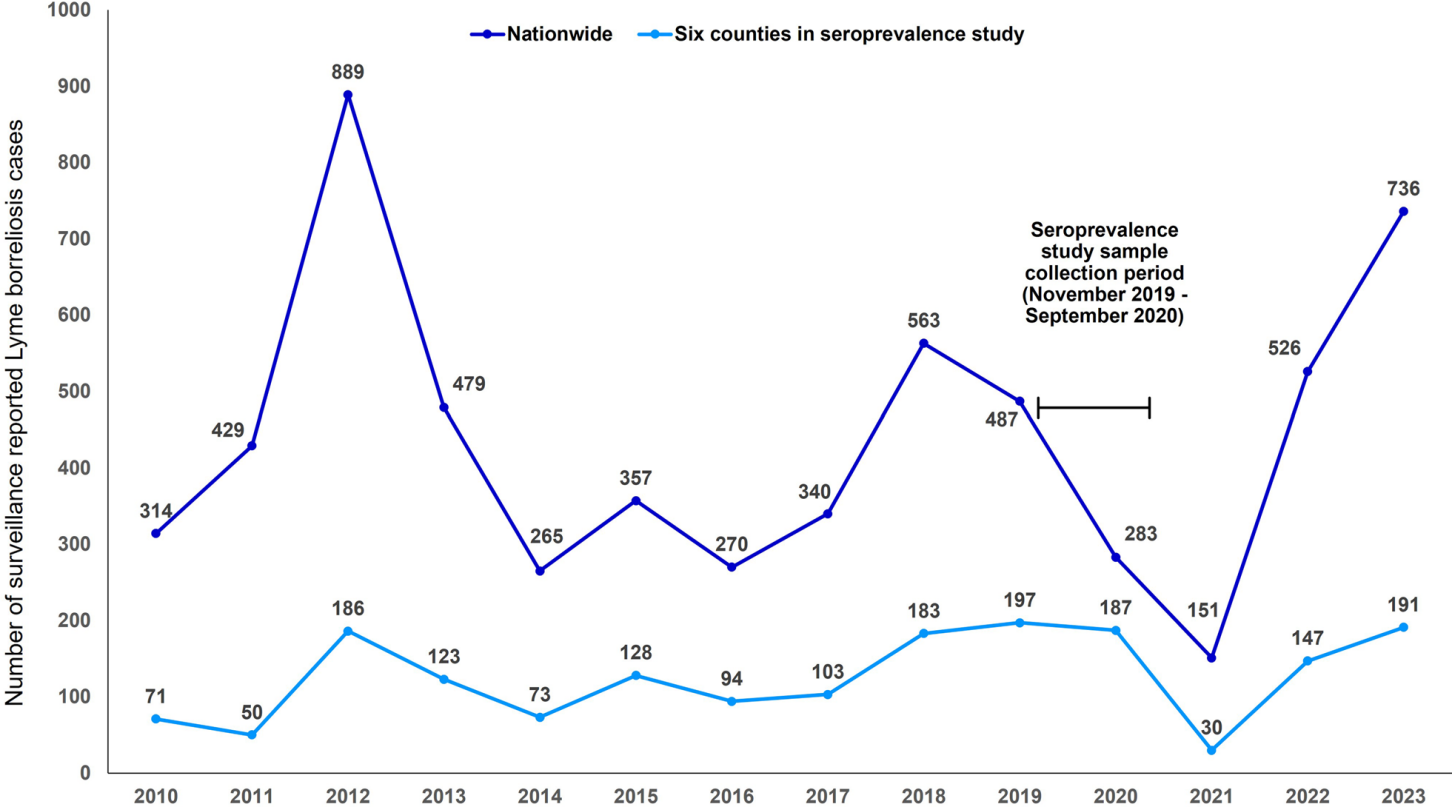
Number of surveillance-reported Lyme borreliosis cases, Romania, 2010–2023

From 2010 to 2023, there were 6089 surveillance-reported LB cases nationwide and 1763 surveillance-reported LB in the six counties where the seroprevalence study was conducted (Fig. 3); the six counties accounted for 28.9% of the surveillance-reported LB cases in Romania. From 2018 to 2023, the incidence (per 100,000 PPY) of symptomatic Bbsl infection in the six countries after adjusting for under-detection was 83.3 in 2018, 89.7 in 2019, 85.1 in 2020, 13.7 in 2021, 66.9 in 2022, and 86.9 in 2023 (Table 2); the incidence was 86.0 in 2018−2020, and 40.3 in 2021−2022.

**Table 2:** Estimates of the incidence (per 100,000 population per year) of symptomatic *Borrelia burgdorferi* sensu lato infection in six counties (Alba, Bistriţa-Năsăud, Cluj, Maramureş, Sălaj, and Satu-Mare) in Romania, 2018−2022

Year	Surveillance-reported LB cases	Incidence of surveillance-reported LB cases	Incidence of Bbsl infection
2018	183	7.9	83.3
2019	197	8.5	89.7
2020	187	8.1	85.1
2021	30	1.3	13.7
2022	147	6.4	66.9
2023	191	8.3	86.9

LB, Lyme borreliosis; Bbsl, *Borrelia burgdorferi* sensu lato

## Discussion

In this analysis, we estimated the incidence of symptomatic Bbsl infection in the counties of Alba, Bistrița-Năsăud, Cluj, Maramureș, Sălaj, and Satu-Mare in north-western and central Romania. The incidence of symptomatic Bbsl infection is notably higher than the incidence of surveillance-reported LB cases. Since estimates of the population-based incidence are essential data for estimating LB burden, the results of this study further our understanding of the LB burden in Romania.

Several other studies have suggested that LB cases are under-detected by the public health surveillance in Romania, but this is the first study to estimate the extent of under-detection [9, 10]. We found that for every surveillance-reported LB case, there were more than 10 cases of symptomatic Bbsl infection. After applying the estimated under-detection multiplier to the public health surveillance data, the highest incidence of symptomatic Bbsl infection in 2018−2023 was 89.7/100,000 population in 2019. The lowest incidence of symptomatic Bbsl infection (13.7/100,000 PPY) was in 2021, which coincided with the COVID-19 pandemic. Impacts of the pandemic may have persisted into 2022 as the incidence in 2022 (66.9/100,000 PPY) was lower than previous years and the incidence in 2023 (86.9/100,000 PPY) was similar to the incidence observed from 2018 to 2020 (86.0/100,000 PPY).

Some of the under-detection of cases of symptomatic Bbsl infection by LB surveillance in Romania is because: (1) the Bbsl infection in some individuals with a symptomatic infection is resolved by their immune system, and there are no subsequent manifestations and they do not seek medical care; or (2) some individuals with a symptomatic Bbsl infection seek medical care but are not diagnosed with LB. In both of these instances, the individuals with a symptomatic Bbsl infection would be unlikely to be reported to LB surveillance because surveillance focuses on reporting of diagnosed LB cases by clinicians (i.e., among patients who seek medical care). Additional studies estimating the incidence of medically-attended LB cases would be helpful in further understanding the LB burden in Romania.

We do not know the extent to which these six counties are representative of other counties in Romania. Although 12% of the Romanian population resided in these six counties in 2020, 29% of the surveillance-reported LB cases in 2010−2023 were from these six counties. If we apply the under-detection multiplier from the six counties to nationwide surveillance data, the estimated incidence of symptomatic Bbsl infection from 2010 to 2023 is 24.1/100,000 PPY. However, we do not know if our estimated under-detection multiplier is representative for all of Romania, or if multipliers in other parts of Romania would be higher or lower than the multiplier in the six counties. Furthermore, the extent of under-detection of symptomatic Bbsl infections by public health surveillance may vary between regions owing to several factors including care-seeking behaviors, clinical awareness of LB, sensitivity and specificity of diagnostic testing, and completeness of disease reporting.

A seroprevalence-based approach has also been used to estimate the incidence of symptomatic Bbsl infection in Finland, Germany, and Poland, but the estimate of the incidence in Romania provides an important contribution to literature as it illuminates differences between the countries [26–28]. The estimate of the incidence of symptomatic Bbsl infection in Romania is lower than in Finland, Germany, and Poland. Reasons for the lower symptomatic LB incidence in Romania may reflect a lower density of *Ixodes* ticks, a lower Bbsl prevalence in ticks, or differences in human behaviors that results in reduced exposure to ticks and infection. The under-detection multiplier in Romania (10.5) is higher than in Finland (2.7) and Poland (5.9), but lower than in Germany (12.0). Differences in under-detection multipliers can be explained by several factors including: (1) Romania only has statutory reporting of LB cases by clinicians whereas the other countries also have statutory reporting by laboratories; (2) Romania may have lower LB awareness, resulting in less care-seeking, undertesting, and underdiagnosis; and (3) Romania may have more non-endemic regions than Finland, Germany, and Poland.

Two key estimates were needed to derive an estimate incidence of symptomatic Bbsl infection: the symptomatic proportion and the duration of persistence of antibodies. On the basis of published literature, we estimated that 37% of individuals infected with Bbsl develop symptoms. As evident by the sensitivity analysis, if a higher proportion of infected individuals are symptomatic, the estimated incidence of symptomatic Bbsl infection increases. On the basis of published literature, we estimated that the duration of persistence of anti-Bbsl IgG antibodies in Bbsl-infected persons is 10 years. As evident by the sensitivity analysis, if the duration is longer, the estimated incidence of symptomatic Bbsl infection decreases. The factors that affect these parameters are not fully elucidated and may vary between countries. For example, antibody persistence may depend on care-seeking and treatment, which could vary by setting. Further research on the estimates of symptomatic proportion and duration of persistence of antibodies are needed to achieve more definitive under-detection multipliers.

Our study has several additional limitations. One limitation is that the seroprevalence study used to estimate under-detection of symptomatic Bbsl infections was conducted among adults. Ideally, we would have estimated under-detection multipliers for several age strata, including children. However, available LB surveillance data in Romania are not stratified by age groups. Thus, we derived the under-detection multiplier using surveillance data for all ages, which required an assumption that the available seroprevalence data were representative for children and adults. However, the seroprevalence of anti-Bbsl IgG antibodies in children in Romania may be lower than that in adults, similar to that reported by studies in Germany [28, 29]. If the seroprevalence is lower in children, there would be fewer incident cases, a smaller under-detection multiplier, and a lower incidence of symptomatic Bbsl infection. Further studies with age-stratified surveillance data would be helpful to better understand age-specific multipliers. 

## Conclusions

In this study, we estimate the incidence of symptomatic Bbsl infection in six counties in Romania for which seroprevalence data are available. This seroprevalence-based approach for estimating the incidence of symptomatic Bbsl infection could be used in other countries that conduct public health surveillance for LB and have representative LB seroprevalence data available. This study represents an important contribution to the understanding of the LB burden in Romania. There is a notably higher incidence of symptomatic Bbsl infection in Romania than is reported in public health surveillance. Additional efforts are needed to strengthen disease prevention and address this important public health problem.

## Data Availability

All data generated or analyzed during this study are included in this published article.

## Abbreviations

B. burgdorferi: *Borrelia burgdorferi*
Bbsl: *Borrelia burgdorferi *sensu lato
EM: Erythema migrans
ELISA: Enzyme-linked immunoassay
LB: Lyme borreliosis
NIPH: National Institute of Public Health
PPY: Population per year
